# Selenoprotein P as an *in vivo* redox regulator: disorders related to its deficiency and excess

**DOI:** 10.3164/jcbn.19-31

**Published:** 2019-11-12

**Authors:** Yoshiro Saito

**Affiliations:** 1 Laboratory of Molecular Biology and Metabolism, Graduate School of Pharmaceutical Sciences, Tohoku University, 6-3, Aoba, Aramaki, Aoba-ku, Sendai 980-8578, Japan

**Keywords:** selenoprotein P, oxidative stress, lipid peroxidation, insulin resistance, insulin secretion, neutralizing antibody

## Abstract

Selenoprotein P (encoded by *SELENOP*) contains the essential trace element selenium in the form of selenocysteine, which is an analog of cysteine that contains selenium instead of sulfur. Selenoprotein P is a major selenium-containing protein in human plasma and is mainly synthesized in the liver. It functions as a selenium-transporter to maintain antioxidative selenoenzymes in several tissues, such as the brain and testis, and plays a pivotal role in selenium-metabolism and antioxidative defense. A decrease of selenoprotein P and selenoproteins causes various dysfunctions related to oxidative stress. On the other hand, recent studies indicate that excess selenoprotein P exacerbates glucose metabolism and promotes type 2 diabetes. This review focuses on the biological functions of selenoprotein P, particularly its role in selenium-metabolism and antioxidative defense. Furthermore, the effects of excess selenoprotein P on glucose metabolism, and resulting diseases are described. The development of a therapeutic agent that targets excess selenoprotein P is discussed.

## Introduction

Selenoprotein P (SeP, encoded by *SELENOP*) is a major selenium (Se)-containing protein in human plasma, and the “P” denotes its presence in plasma.^(1–3)^ SeP is synthesized mainly in the liver and secreted to extracellular fluid. SeP contains the essential trace element Se in the form of selenocysteine (Sec), which is an analog of cysteine that contains Se instead of sulfur.^(4–6)^ Twenty-five kinds of selenoproteins have been discovered in humans, including five types of glutathione peroxidases (GPxs) and three types of thioredoxin reductases (TrxRs), which play a pivotal role in antioxidative defense.^(7–9)^ SeP functions as a Se-transporter to maintain antioxidative selenoenzymes in several tissues, such as the brain and testis, and plays a crucial role in the metabolism of Se and antioxidative defense.^(10–12)^ A decrease of SeP and selenoproteins causes various dysfunction related to oxidative stress;^(13–16)^ however, recent studies indicate that excess SeP exacerbates glucose metabolism and promotes type 2 diabetes.^(17–19)^ This review focuses on the biological functions of SeP, particularly its role in Se-metabolism and antioxidative defense. Furthermore, disorders induced by excess SeP that affect the glucose metabolism are summarized, and the development of a therapeutic agent that targets excess SeP is discussed here.

## Structure and Function of SeP

**Fig. 1:**
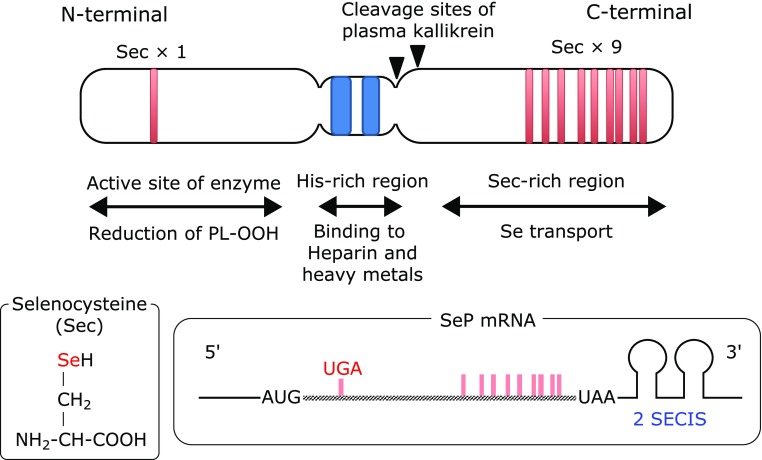
Domain structure of human selenoprotein P. SECIS, Sec insertion sequence.

SeP contains ten Sec residues per polypeptide, which are encoded by the UGA stop codon (Fig. 1). SeP is a unique selenoprotein with multiple Sec residues, while other selenoproteins only have one or two Sec residues.^(20,21)^ The distribution of Sec residues in SeP is biased; one Sec is located in the N-terminal region, and the other nine are located in the C-terminal region (Fig. 1). SeP is a multifunctional protein, possessing GPx-like enzyme activity to reduce phospholipid hydroperoxide in the presence of glutathione and Se-transport activity to effectively supply Se to cells.^(22–25)^ Human SeP is a substrate of the serine protease plasma kallikrein, which generates N- and C-terminal fragments.^(11)^ Based on the biological function of these fragments, domain structure of SeP is proposed;^(11)^ Sec in the N-terminal region is the active site of the enzyme that reduces phospholipid hydroperoxide, while the Sec-rich C-terminal region functions as a Se-transporter (Fig. 1). A His-rich region that contains consecutive His residues is located in the middle of SeP. SeP possesses heparin-binding properties, and a typical heparin-binding motif XBBXB (B: a basic amino acid), is found in the His-rich region.^(26–28)^ SeP binds heavy metals such as copper and cadmium, and its consecutive His residues function as a natural, high-affinity His-tag binding site for nickel-nitrilotriacetic acid (Ni-NTA) agarose.^(29–31)^ SeP is also a major methylmercury-binding protein in plasma, suggesting that it plays a role in the detoxication of heavy metals.^(32–34)^

## Significant Role of SeP in Se-metabolism *in vivo*

**Fig. 2:**
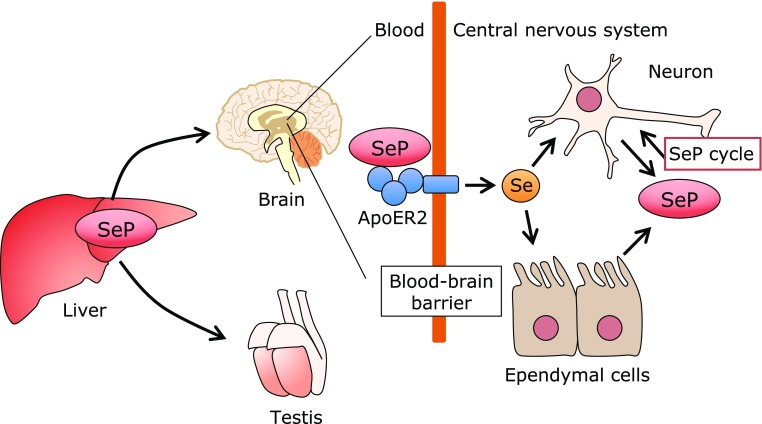
Selenium transport mechanism via selenoprotein P. Selenoprotein P (SeP) synthesized in the liver is preferentially incorporated by the brain and testis via SeP-receptor ApoER2/LRP8. In the brain, several cells such as neurons and ependymal cells synthesize SeP, which contributes to maintain Se and selenoprotein levels in the brain.

Data regarding the significant role of SeP in Se-metabolism *in vivo* have been obtained from SeP knockout (KO) mice.^(35–39)^ SeP KO mice can survive on a normal chow diet containing 0.4 mg Se/kg, which contains enough Se to maintain maximum selenoprotein levels in wild type (WT) mice.^(40)^ However, SeP KO mice cannot survive on a Se-deficient diet, indicating the significant role of SeP as a Se-transporter when Se supply is limited. On the other hand, when SeP KO mice are on a normal chow diet with enough Se, Se levels are noticeably low in their brain and testis, which demonstrates the essential role of SeP in delivering Se to these tissues (Fig. 2). One obvious phenotype of SeP KO mice is the failure of spermatogenesis, which cannot be reversed by Se supplementation. Another phenotype is neurological dysfunction when the supply of Se is limited (<0.1 mg Se/kg diet, which satisfies the Se requirement of WT mice); SeP KO mice develop progressive spasticity that requires euthanasia.^(41–43)^ This neurological dysfunction in SeP KO mice is prevented by a chow diet with more than 0.25 mg Se/kg. Three kinds of SeP receptors have been identified, namely apolipoprotein E receptor 2 (ApoER2)/low-density lipoprotein receptor-related protein 8 (LRP8), megalin, and LRP1, which belong to the low-density lipoprotein receptor family.^(44–46)^ Similar phenotypes have been reported in ApoER2 KO mice, suggesting the importance of receptor-mediated uptake of Se in the brain and testis.^(47)^ Similar phenotypes, but with a later onset or less severe implications, in the brain and testis have been reported in *Sepp1*^Δ240–361^ mice, in which the Sec-rich C-terminal domain of SeP had been deleted.^(41)^ The interaction between the C-terminal domain of SeP and the YWTD β-propeller domain of ApoER2 has been reported, and the importance of this interaction, particularly in maintaining Se levels in the brain and testis, has been manifested in the phenotypes of these KO mice (Fig. 2).^(48)^ On the other hand, on the normal diet that contains enough Se, Se levels in other tissues of SeP KO mice, expect for the brain and testis, do not differ greatly from those in WT mice, and, in line with these Se levels, other obvious phenotypes do not appear in these KO mice. These findings suggest that SeP-dependent and SeP-independent Se-transport systems exist *in vivo*. Approximately half of the Se in human plasma is derived from SeP, while extracellular GPx (GPx3) contains 20% of Se.^(10)^ The remaining Se might be present as low molecular weight Se and selenomethionine, which are mainly associated with and/or contained by albumin. In fact, GPx3, selenomethionine, and albumin function as a source of Se for the synthesis of cellular selenoproteins.^(10)^

The organ in which SeP is mostly synthesized is the liver, as described above, but SeP-expressing cells are found in several other tissues. Based on blood SeP concentrations of liver-specific SeP KO mice, it is estimated that 60% of SeP is derived from the liver and the remaining 40% from other tissues.^(49,50)^ SeP expression in the brain is important for maintaining Se and selenoprotein levels, and Se concentrations in the brain are preserved in liver specific-SeP KO mice (Fig. 2). SeP expression has been reported in several cell types of the brain, including neurons, and ependymal cells that are responsible for cerebrospinal fluid production.^(51,52)^ Presumably, SeP synthesized in the brain is incorporated to other brain cells and used to synthesize selenoproteins, which help to maintain Se concentrations in the brain (Fig. 2). This system is called the SeP cycle, and it retains selenoproteins in several tissues and cells.

## Increase of SeP Levels under High Glucose and High Fat Conditions

**Fig. 3:**
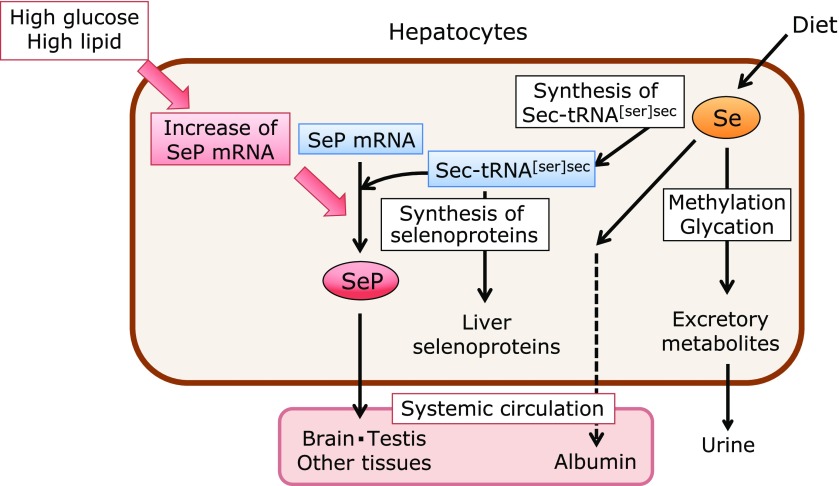
Selenium metabolism in the liver. Diet-derived Se is used for Sec synthesis or excreted following methylation/glycosylation. Se in the Sec synthesis pathway is used for the synthesis of liver selenoproteins or SeP, which enter the systemic circulation. In addition, small Se-containing compounds bound to albumin and/or selenomethionine also enter the systemic circulation. High glucose and high lipid diets increase SeP mRNA and enhance the synthesis of SeP when enough Sec-tRNA^[ser]sec^ is available.

Se is an essential trace element, and its deficiency causes numerous dysfunctions due to the decrease of selenoproteins.^(53–55)^ However, recent evidence shows an increase of SeP in type 2 diabetes patients, and excess SeP levels have undesirable effects on the glucose metabolism.^(17–19,46,56–58)^ This indicates that increased SeP levels are a significant therapeutic target for this lifestyle-related disease. The increase of SeP in diabetic patients has been discovered by comprehensive gene expression analysis of mRNA samples obtained from liver biopsies of diabetic patients.^(17)^ A significantly positive correlation between SeP expression levels and biomarkers of diabetes, such as fasting glucose and blood glucose levels after administrations of a glucose tolerance test, has been shown.^(17,57–60)^ To increase SeP levels, enough Se intake is necessary; however, this might not be sufficient. Normal mice chow contains 0.4 mg Se/kg, which causes neither an increase in SeP levels nor high blood glucose levels in normal mice. In contrast, high fat, high sucrose chows containing 0.2 mg Se/kg induce diabetes in mice and increase SeP levels.^(17,18)^ Therefore, it is assumed that SeP levels increase when both high energy food sources and enough Se are available, which allows SeP mRNA levels to increase and to synthesize SeP proteins (Fig. 3). Accordingly, limiting the Se intake may be effective in diabetic patients with high SeP levels; however, this might be difficult, because numerous foods contain trace amounts of Se. In a large-scale clinical trial in humans, it has been reported that the prolonged intake of Se supplements (200 µg/day) increases the risk of type 2 diabetes; however, it is noteworthy that this observation is limited to a population that has high Se baseline blood levels.^(56)^

Metformin is a widely used drug to treat diabetes and lowers SeP expression in the liver.^(61–63)^ Metformin activates AMP-activated kinase (AMPK), and activated AMPK subsequently inactivates the transcription factor FoxO3a and decreases SeP mRNA levels. In the promotor region of SeP, FoxO-binding sites have been identified, and the AMPK-FoxO3 axis is an important pathway that links energy metabolism and SeP expression. In addition, eicosapentaenoic acid (EPA), a major component of ω-3 polyunsaturated fatty acids (PUFAs) contained in fish oil, has been reported to decrease SeP expression in H4IIEC3 hepatocytes.^(64–66)^ EPA is used to improve lipid metabolism and has beneficial effects in the treatment of type 2 diabetes. EPA has been known to activate AMPK; however, EPA decreases the binding of sterol regulatory element-binding protein-1c (SREBP-1c) to the SeP promoter region and lowers SeP levels. These results suggest that SeP levels are transcriptionally regulated by FoxO3a and SREBP-1 activation, which are closely related to glucose and lipid metabolism.

## Negative Effects of Increased SeP on Insulin Resistance and Insulin Secretion

Recent studies show that increased SeP in type 2 diabetic patients worsens glucose metabolism via the impairment of insulin resistance and insulin secretion.^(17,18)^ Injection of human SeP protein at a concentration that reflects the increment of SeP concentrations in diabetic patients inhibited insulin signal transduction in normal mice and induced hyperglycemia during the glucose tolerance test.^(17,18)^ SeP KO mice exhibited the resistance against high fat, high sucrose diet-induced hyperglycemia or insulin resistance. Excess SeP levels induced insulin resistance in primary hepatocytes and C2C12 myocytes, a model of skeletal muscles, via the inhibition of AMPK activity.^(17)^ AMPK is activated not only by AMP but also by several factors including reactive oxygen species (ROS).^(67–69)^ It is known that ROS generated during exercise activate AMPK, increase peroxisome proliferator-activated receptor gamma coactivator 1-α (PGC-1α), and exert health-promoting effects.^(70–72)^ These positive effects of ROS generated by exercise are reduced by excess SeP, resulting in a decrease of AMPK phosphorylation and health-promoting effects. This new clinical concept is called “exercise resistance.”^(46)^ SeP is incorporated in the skeletal muscle by LRP1, and the SeP-LRP1 axis is an important regulator of exercise resistance. Furthermore, it has been reported that increased amounts of circulating SeP levels predicted the ineffectiveness of training on the endurance capacity in humans, suggesting that the SeP-LRP1 axis is a significant target for the treatment of diseases associated with a sedentary lifestyle.^(46)^

**Fig. 4:**
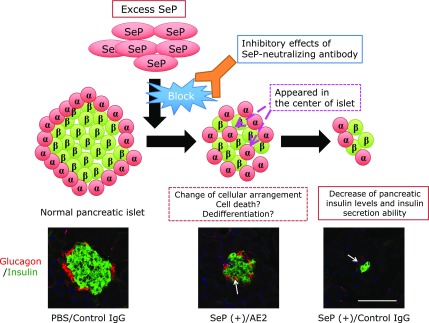
Pancreatic β-cell dysfunction induced by excess selenoprotein P. Increased SeP is incorporated by the pancreas, decreases the insulin levels in β cells, and reduces insulin secretion triggered by a high glucose stimulus. Immunohistochemical analysis of the pancreas of SeP- and neutralizing antibody AE2-administered mice indicated that excess SeP alters the cellular distribution of islets. The histochemical analysis is shown in the lower panel: anti-insulin Ab (green, indicative of β-cells) and anti-glucagon Ab (red, indicative of α-cells). Scale bars = 100 µm.

It has been shown that excess SeP impairs the function of pancreatic β-cells and decreases insulin secretion.^(18)^ When excess amounts of SeP are added to MIN6 cells, a model for pancreatic β-cells, cellular insulin levels and high glucose-induced insulin secretion decrease significantly. Excess SeP also inhibits the insulin secretion of rat primary pancreatic islets; equally excessive amounts of selenocystine showed similar inhibitory effects on insulin secretion, suggesting a relation with the Se-transport activity of SeP.^(18)^ The impairing effects of excess SeP on pancreatic function have been observed in *in vivo* experiments, and the injection of purified human SeP protein resulted in the decrease of pancreatic insulin levels, area of islets, and glucose-induced insulin secretion (Fig. 4).^(18)^ SeP injection resulted in a decrease of not only β-cells but also α-cells in the pancreas, which might be accompanied by a rearrangement of the position of these cells in the pancreatic island (Fig. 4). The rearrangement and decrease of both α- and β-cells has been observed in the animal diabetes model and in humans.^(73–75)^ Recently, it has been shown that SeP levels are negatively correlated with the insulinogenic index, an indicator of insulin secretion,^(57)^ suggesting that excess SeP is a considerable therapeutic target to protect pancreas function in patients with type 2 diabetes.

It is also notable that excess SeP impairs angiogenesis by inhibiting vascular endothelial growth factor (VEGF) signaling in vascular endothelial cells.^(76)^ This is a hallmark of vascular complications in type 2 diabetes, and ROS generated by VEGF stimuli are important for the phosphorylation of VEGF receptor 2 (VEGFR2) and extracellular signal-regulated kinase 1/2 (ERK1/2) in human umbilical vein endothelial cells (HUVECs). Treatment with excess SeP inhibited VEGF-stimulated proliferation and the phosphorylation of VEGFR2 and ERK2 in HUVECs, which was significantly improved by the addition of buthionine sulphoximine (BSO), an inhibitor of glutathione synthesis.^(76)^ Therefore, the adverse effects of increased SeP in type 2 diabetes are diverse and might be related to vascular complications.

## Significance of SeP Expression in Pulmonary Arterial Hypertension

Recently, it has been described that the increased expression of SeP in pulmonary artery smooth muscle cells (PASMCs) forming lesions of pulmonary arterial hypertension (PAH).^(77)^ PAH-PASMCs are proliferative compared with normal PASMCs, and the pulmonary artery is constricted/occluded by abnormal proliferation of PAH-PASMCs, which induces PAH with right heart failure. High expression of SeP in PAH-PASMCs has been discovered by comprehensive gene and protein expression analysis of PAH-PASMCs and control PASMCs. PASMC-specific SeP KO mice and mice treated with SeP-lowering drugs showed improvement of PAH symptoms; therefore, it is suggested that increased expression of SeP in PAH-PASMCs is a significant mediator of lesion formation in PAH.^(77)^ The decrease of SeP expression by SeP-siRNA treatment inhibits the proliferation of PAH-PASMCs, and these effects are mediated by the SeP-receptor ApoER2.^(77)^ This observation reminds the SeP cycle described above; however, interestingly, the proliferative effects of SeP were not explained by Se-transport activity, namely the addition of selenocystine did not reproduced this effect of SeP, and the proliferation-promoting effects were observed by the overexpression of the mutant in which all Secs were substituted with Cys.^(77)^ Thus, the proliferative effects of increased SeP in PAH-PASMCs are considered to be mediated by autocrine and/or paracrine stimuli from the SeP-receptor ApoER2. Se-independent biological effects of SeP have been recently described in a study on PAH, and it is interesting to speculate about the possibilities to relate not only to PAH, but also other physiological and/or pathological events.

## Therapeutic Strategies to Treat Increased SeP

**Fig. 5:**
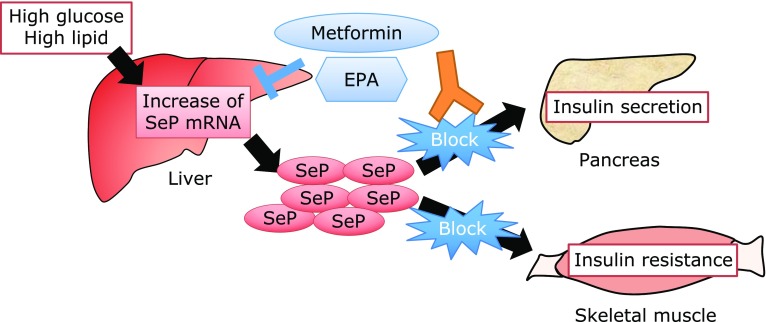
Therapeutic strategies that target increased SeP in related diseases.

These lines of evidence indicate that increased SeP is a significant therapeutic target for type 2 diabetes and its vascular complications. The strategy to treat excess SeP is shown in Fig. 5. Metformin and EPA decrease SeP mRNA expression.^(61,64)^ However, these diabetes drugs are not effective against PAH-PASMC proliferation, which suggests other molecular mechanisms that increase SeP expression in PAH.^(77)^ To inhibit the SeP expression in PAH-PASMCs, sanguinarine, a plant alkaloid, has been identified by high-throughput screening of 3,336 compounds. Sanguinarine reduced SeP expression and proliferation in PAH-PASMCs and ameliorated PAH in animal models.^(77)^

SeP-neutralizing antibodies have been developed to inhibit the Se-transport activity of SeP *in vitro* and *in vivo*, and two SeP-neutralizing monoclonal antibodies, namely clones AE2 and AA3, have been identified.^(18)^ The epitope of AE2 is the N-terminal side of the His-rich region, while that of AA3 is the C-terminal region, which is Sec-rich.^(78)^ The C-terminal region of SeP has been reported to bind to the β-propeller domain of ApoER2, while detail binding sites are not fully elucidated in other receptors. Several variants of ApoER2 are reported, and their efficiency of Se-transport activity varies depending on the tissues and type of cells. The significant inhibitory effects of AE2 and heparin sulfate on SeP binding and Se-transport activity suggests the importance of interaction between His-rich region and carbohydrate moiety of the cells.^(18)^ SeP-neutralizing antibodies suppressed the incorporation of injected SeP in the skeletal muscle and pancreas, and improved insulin resistance and insulin secretion that had been impeded by the injection of excess SeP (Fig. 5). SeP-neutralizing antibodies are effective against diabetes in model mice, such as KKAy mice and mice fed with a high fat and high sucrose diet, in which SeP levels are increased.^(18)^ In addition to improved insulin resistance and insulin secretion, SeP-neutralizing antibodies significantly decreased total cholesterol and triglycerides in the liver of KKAy mice, indicating the diverse beneficial effects of SeP-targeting reagents. Selenoprotein I is identified as ethanolamine phosphotransferase 1 (EPT1), playing a significant role in phospholipid synthesis.^(79,80)^ How Se and/or SeP levels regulate lipid metabolism is not fully elucidated, which makes them a potential new target for therapeutic agents.

## Conclusions

This brief review focuses on the biological functions of SeP and its implications in several diseases. The change of SeP levels significantly affects the homeostasis of the whole body, and its dysregulation is related to several diseases. These findings strongly support the necessity of SeP determination at clinical sites. Furthermore, they suggest the usefulness of establishing tailor-made treatments based on SeP levels.

## Abbreviations

AMPK: AMP-activated kinase
ApoER2: apolipoprotein E receptor 2
BSO: buthionine sulphoximine
EPA: eicosapentaenoic acid
ERK: extracellular signal-regulated kinase
GPx: glutathione peroxidase
HUVEC: human umbilical vein endothelial cell
LRP: low-density lipoprotein receptor-related protein
PAH: pulmonary arterial hypertension
PASMC: pulmonary artery smooth muscle cell
PGC-1α: peroxisome proliferator-activated receptor gamma coactivator 1-α
ROS: reactive oxygen species
Se: selenium
Sec: selenocysteine
SeP: selenoprotein P
SREBP: sterol regulatory element-binding protein
TrxR: thioredoxin reductase
VEGF: vascular endothelial growth factor
VEGFR2: VEGF receptor 2
